# Dissociated Representations of Pleasant and Unpleasant Olfacto-Trigeminal Mixtures: An fMRI Study

**DOI:** 10.1371/journal.pone.0038358

**Published:** 2012-06-12

**Authors:** Moustafa Bensafi, Emilia Iannilli, Johan Poncelet, Han-Seok Seo, Johannes Gerber, Catherine Rouby, Thomas Hummel

**Affiliations:** 1 Lyon Neuroscience Research Center, CNRS, UMR5292, University Lyon, Lyon, France; 2 Department of Otorhinolaryngology, Smell & Taste Clinic, University of Dresden Medical School, Dresden, Germany; 3 Department of Neuroradiology, University of Dresden Medical School, Dresden, Germany; French National Centre for Scientific Research, France

## Abstract

How the pleasantness of chemosensory stimuli such as odorants or intranasal trigeminal compounds is processed in the human brain has been the focus of considerable recent interest. Yet, so far, only the unimodal form of this hedonic processing has been explored, and not its bimodal form during crossmodal integration of olfactory and trigeminal stimuli. The main purpose of the present study was to investigate this question. To this end, functional magnetic resonance imaging (fMRI) was used in an experiment comparing brain activation related to a pleasant and a relatively unpleasant olfacto-trigeminal mixture, and to their individual components (CO_2_ alone, Orange alone, Rose alone). Results revealed first common neural activity patterns in response to both mixtures in a number of regions: notably the superior temporal gyrus and the caudate nucleus. Common activations were also observed in the insula, although the pleasant mixture activated the right insula whereas the unpleasant mixture activated the left insula. However, specific activations were observed in anterior cingulate gyrus and the ventral tegmental area only during the perception of the pleasant mixture. These findings emphasized for the firs time the involvement of the latter structures in processing of pleasantness during crossmodal integration of chemosensory stimuli.

## Introduction

Perception of intranasal chemical stimuli is not dependent on a single sensory system but is related to multiple sensations, mediated principally by interaction between the olfactory and trigeminal systems [1], [2], [3], [4], [5], [6]. Whereas olfaction is involved in qualitative discrimination of odors, the trigeminal system conveys information about sensations such as cooling, burning, irritation and pain [7]. Furthermore, both odorants and trigeminal compounds evoke pleasant or unpleasant affects. One important question raised by psychologists and neurobiologists in the field during the last decade concerns the cerebral correlates of such pleasantness perception induced by these two types of chemosensory stimuli. However, whereas odorants and trigeminal compounds provide both synergetic and complementary information about the hedonic aspect of perceived objects, little is known about the neural basis that accompanies the perception of pleasantness during their crossmodal integration. So far, only the unimodal functioning of the neural substrate of this hedonic processing has been explored, notably in the olfactory modality. For example, pleasant and unpleasant odors induced distinct patterns of neural activity in primary olfactory areas [8], [9], [10], [11] and amygdala [12] and also in secondary and tertiary areas such as the orbito-frontal cortex (OFC) [13], [14], [15], the thalamus [16] and the cingulate gyrus [17]. Understanding whether the pleasantness of chemosensory stimuli is processed within the same brain network during crossmodal activation is a central question in neuroscience because, in everyday life, coordinated interplay between olfactory and trigeminal systems is frequent. For example, when one drinks orangeade, the olfactory system will detect the smell of orange and the trigeminal system will detect carbon dioxide; when one smells mint, the trigeminal system will detect the characteristic freshness of the mint odor. The main purpose of the present study was to investigate this question using functional magnetic resonance imaging (fMRI).

To this end, 23 participants were exposed to two different bimodal mixture (CO_2_ combined with either the smell of orange or the smell of rose) and to the individual components of the mixtures (CO_2_, smell of orange and smell of rose). In all conditions, participants were required to identify the stimulus, and evaluate its intensity and pleasantness. Inter-individual variations in hedonic perception are common in chemosensory perception [18], [19], [20] and our results confirm this observation: whereas fifteen participants found the [CO_2_+Orange] more pleasant than [CO_2_+Rose], 6 showed the opposite pattern ([CO_2_+Rose] more pleasant than [CO_2_+Orange]), and 2 participants did not show hedonic differences between mixtures. This variation was thus taken into account and brain activations related to the pleasant and the relatively unpleasant mixture according to individual subject’s ratings (whatever the mixture quality, namely [CO_2_+Orange] or [CO_2_+Rose]) were compared.

## Results

### The Pleasant and the Relatively Unpleasant Mixtures Induced Different Neural Activations

**Figure 1:**
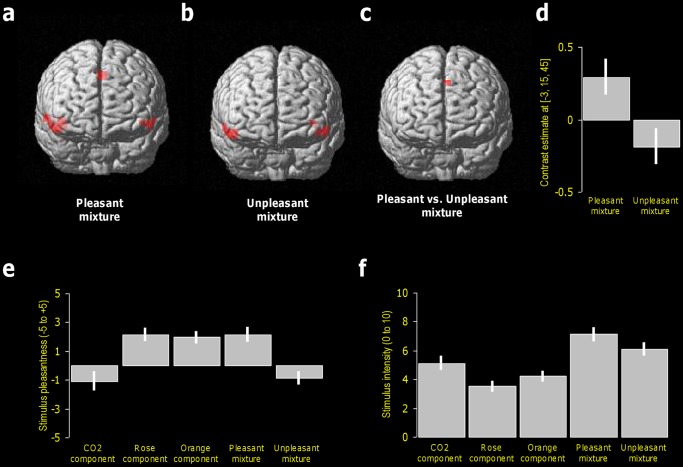
Perceptual ratings and brain activations. (a) Three-D view of fMRI activation maps showing activation to the pleasant mixture after subtraction of activation to their individual components: brain responses were seen in the superior temporal gyrus, insula and cingulate gyrus. (b) Three-D view of fMRI activation maps showing activation to the unpleasant mixture after subtraction of activation to their individual components: brain responses were seen in the superior temporal gyrus and insula, but not in cingulate gyrus. (c) Differential activation patterns, showing activation to ([pleasant mixture] vs. [unpleasant mixture]). Brain responses were seen in the cingulate gyrus. (d) Contrast estimates at voxel coordinates [−3, 15, 45] in cingulate cortex for each mixture condition. (e) Compound pleasantness ratings. (f) Compound intensity ratings. Bars represent SEM.

To identify the neural substrates involved in crossmodal integration of pleasantness, activation in response to the pleasant mixture was compared to that for its individual components. MNI coordinates (x, y, z) of activated brain areas and statistical t values are presented in parentheses. The results revealed significant activations in the insula (39, 18, −9, t = 7.21), the superior temporal gyrus (48, 15, −12, t = 7.92; 57, 9, −6, t = 7.19; −51, 15, −9, t = 7.73), the caudate nucleus (12, −3, 15, t = 7.61) and the posterior part of the anterior cingulate gyrus (0, 15, 45; t = 8.25) (Figure 1a; Table 1). Moreover, to ascertain regions of the brain responding preferentially to crossmodal integration of unpleasantness, we compared brain activation in the relatively unpleasant mixture condition to that resulting from its individual components. An activation was observed in the insula (−36, 18, 0, t = 6.72), superior temporal gyrus (48, 15, −12, t = 7.68; −51, 15, −9, t = 7.10; 57, 9, −6, t = 6.92), and the caudate nucleus (12, −3, 18, t = 7.14) (Figure 1b; Table 1).

To further examine the differential effect of the mixtures, the activation induced by the pleasant mixture minus its components was compared to that resulting from the relatively unpleasant mixture minus its components. A significant activation was observed in the posterior part of the anterior cingulate gyrus (−3, 15, 45, t = 3.32) (Figures 1c, 1d) and in the ventral tegmental area bordering the pons (3, −24, −24, t = 3.60). In turn, the opposite contrast did not show any significant activation.

**Table 1:** Activation to [pleasant mixture] and [unpleasant mixture] after subtraction of activation to their individual components and activation to [pleasant mixture] vs.[unpleasant mixture].

	K	t value	x	y	z	Brain areas
[Pleasant mixture] vs. [Individual components]	21	8.25	0	15	45	Cingulate gyrus
	40	7.92	48	15	−12	Superior temporal gyrus
		7.21	39	18	−9	Insula
		7.19	57	9	−6	Superior temporal gyrus
	18	7.73	−51	15	−9	Superior temporal gyrus
	5	7.61	12	−3	15	Caudate nucleus
						
[Unpleasant mixture] vs. [Individual components]	18	7.68	48	15	−12	Superior temporal gyrus
	4	7.14	12	−3	18	Caudate nucleus
	10	7.10	−51	15	−9	Superior temporal gyrus
	3	6.92	57	9	−6	Superior temporal gyrus
	3	6.72	−36	18	0	Insula
[Pleasant mixture] vs. [Unpleasant mixture]	3	3.32	−3	15	45	Anterior cingulate gyrus
	4	3.60	3	−24	−24	Ventral tegmental area/pons

K is the cluster size. Statistical t values are presented. MNI coordinates of activated brain areas are presented in x, y, and z.

### CO_2_ did not Suppress the Perception of Odors in the Mixtures

The stimulus identification results revealed no significant difference between any of the experimental conditions (Friedman Test, X^2^ = 0.333 p = .98), suggesting that in the bimodal mixtures CO_2_ did not suppress the perception of the odor of rose or orange: among 21 subjects included in the analysis (see methods), the number of subjects with correct identification was: [CO_2_] = 16, [Rose] = 17, [Orange] = 15, unpleasant mixture = 15, pleasant mixture = 16.

### Effects on Pleasantness and Intensity

To assess differences in pleasantness between stimuli, an ANOVA with compounds ([CO_2_], [Rose], [Orange], [Pleasant mixture], [Unpleasant mixture]) as a within-subjects factor was performed. A significant effect of compound was observed (F[4,76] = 8.776, p<0.0001), indicating that 1) the relatively unpleasant mixture and [CO_2_] did not differ in pleasantness (p = .99), 2) the pleasant mixture, [Rose] and [Orange] did not differ in pleasantness (p>.98 in all three comparisons), 3) the pleasant mixture, [Rose] and [Orange] were all three significantly more pleasant than both the relatively unpleasant mixture and [CO_2_] (at least p<.0006 in all comparisons) (Figure 1e).

For intensity ratings, a significant effect of compound was noted (F[4,76] = 13.863, p<0.001), and post-hoc tests revealed that 1) no difference in intensity appeared between the pleasant mixture and the relatively unpleasant mixture (p = .32), 2) intensity differed between [CO_2_] and [Rose] (p = .03), but not between [Rose] and [Orange] (p = .63) and [CO_2_] and [Orange] (p = .47), 3) the pleasant mixture was rated as more intense than CO2, Rose, Orange (p<.005 in all cases), and 4) that the relatively unpleasant mixture was rated as more intense than Rose (p<.0002) and Orange (p = .0002) (Figure 1f).

Nevertheless, to correct for the potential influence of stimulus intensity on pleasantness ratings between the two mixtures, difference in compound intensity between the pleasant mixture and the relatively unpleasant mixture was used as covariate in an ANCOVA with mixture pleasantness as the dependent variable. When the effect of intensity was factored out, the ANCOVA revealed a strongly significant effect of mixtures on odor pleasantness (F[1], [19] = 28.854, p<.0001). In sum, differences in pleasantness between the two mixtures cannot be explained by differences in intensity.

## Discussion

The aim of the present study was to explore the neural substrate involved in the perception of pleasantness during crossmodal integration of intranasal stimuli. A first result of interest was that common neural activity patterns were observed in response to the pleasant and the relatively unpleasant mixtures in a number of regions. The perception of the two mixtures was associated with activation in the superior temporal gyrus. Interestingly, this region is known to be involved in cross-modal binding processing in the auditory and visual systems [21] and our results emphasized its role during cross-modal integration of chemosensory stimuli. Moreover, both mixtures induced activation in the insula and in the caudate nucleus, replicating previous findings showing a brain response of the former area during perception of an olfacto-trigeminal mixture [22] and in integration of multisensory information for the latter brain structure [23]. Insula activation in response to both the emotionally positive and negative mixtures is also consistent with previous findings of increased insular activity during perception of pleasant and unpleasant tastes [24]. An interesting aspect of the insular activity is the hemispheric asymmetry observed according to pleasantness: whereas the pleasant mixture induced activation of the right insula, the unpleasant mixture induced activity in the left insula. This hemispheric dissociation contributes to the ongoing debate dealing with brain lateralization of olfactory emotions: whereas some authors propose an involvement of the right hemisphere in withdrawal behavior and of the left hemisphere in approach behavior [25] others propose a right hemisphere specialization for pleasant stimuli versus left hemisphere for unpleasant stimuli [13], [16]. Our results support the latter model and helps to explain previous observations on hemispheric differences of amplitudes of event-related potentials in response to pleasant and unpleasant olfactory stimuli [26].

Another major result of the present study was the specific activation seen during the perception of the pleasant mixture: notably in the cingulate gyrus. Activity in this brain area is usually observed in response to chemosensory stimuli [27], [28], [29], [30]. An investigation in humans proposed the cingulate cortex as a multi-integrative structure in processing chemosensory stimuli: for example, Small et al. showed increased activity in this brain region when a tastant and an odorant were concurrently perceived [31]. Anatomically, cyto-architectural studies of the cingulate gyrus support a multiple-region model rather than the classical two-division model proposed by Brodmann [32]. The functioning of these sub-regions is not homogeneous and the different parts of the cingulate cortex are not equally involved in emotion processing. A meta-analysis of several studies exploring neural activation in the cingulate cortex in response to emotional stimuli proposed that emotions such as happiness predominantly activate the posterior part of anterior cingulate cortex (see [32] for a review). The present findings are in line with the above results, highlighting a role of this brain area in processing stimulus pleasantness. They also highlight a role of this area in binding olfactory and trigeminal representations of environmental objects. It is worth to note that a previous study by Small et al. showed an activation of the left and right posterior part of the anterior cingulate gyrus in response to an unpleasant taste [24]. However, these activations were either more caudal or more lateral (MNI coordinates: −18, −6, 39; 15, 6, 38) compared to those observed in our study (MNI coordinates: 0, 15, 45; −3, 15, 45).

Although the present study provides evidence for modulation of the cingulate cortex by pleasantness, some neural activation seen during the perception of the pleasant mixture warrants discussion. Indeed, another particular feature of the present findings was the ventral tegmental area (VTA) activation in response to the pleasant mixture vs. the relatively unpleasant mixture. These results are in line with psychobiological theory of positive affect [33] that highlights a role of the VTA in reward processing. Interestingly, in this model, positive affect is associated with increased dopamine release from the VTA which may alter processing in structures receiving direct projections from the VTA, including both primary olfactory structures and cingulate cortex.

One question that may be raised by these findings is why a mixture comprising a pleasant odor (rose or orange depending on the subject) and a painful trigeminal stimulus (intranasal CO_2_) was rated as pleasant? A plausible explanation is related to subjects’ prior experience of the simultaneous presentation of the two types of stimuli: intranasal CO_2_ is frequently mixed with certain other olfactory stimuli. Even when one unimodal stimulus (here, CO_2_) arouses a sensation of pain, this intrinsically painful feature becomes part of the integrated percept of a familiar object or food. As suggested by Rozin et al. [34], the memory representation of this food may thus inhibit the pain or warning value of the trigeminal input (CO_2_ here), and even make it desirable.

In conclusions, our study offers new insights into the affective processing of chemosensory stimuli by emphasizing for the first time the involvement of the cingulate cortex and the midbrain during crossmodal integration of pleasantness of chemosensory stimuli.

## Material and Methods

### Ethics Statement

The experimental procedure was explained in great detail to the subjects, who provided written consent prior to participation. The study was conducted according to the Declaration of Helsinki and was approved by the ethical committee of the University of Dresden.

### Subjects

Among 23 subjects that participated to the study, 21 were retained in the final analysis (2 subjects who did not show hedonic differences between the two mixtures were discarded from the analysis). Subjects were right-handed volunteers, averaging 23 years of age (23.57±2.08 years; 6 men). They received 20 Euros for participation. The recording procedure was explained in great detail to the subjects, who provided written consent prior to participation. Instructions consisted in an explanation of the experimental design that includes functional and anatomical sessions. In both sessions, they were instructed to not move. For functional sessions, they were instructed to breathe through the mouth without concomitant nasal airflow (velopharyngeal closure, see “Stimulus delivery” section” below). Detailed medical history combined with ENT examination of the nasal cavity using nasal endoscopy technique and odor perception assessment by the “Sniffin’ Sticks” test [35] ascertained that subjects were in good health and had normal sense of smell.

### Stimulus Delivery

A Burghart OM6b pulsed olfactometer was used to deliver the 5 stimuli. It allows application of rectangular-shaped chemical stimuli with controlled stimulus onset. Mechanical stimulation is avoided by embedding stimuli in a constant flow of odorless, humidified air of controlled temperature (80% relative humidity; total flow 6 L/min; 36°C) [36]. Prior to the functional experiment, subjects were trained in lab to breathe through the mouth without concomitant nasal airflow (velopharyngeal closure [36]), to avoid respiratory airflow in the nasal cavity during chemosensory stimulation. A thermally insulated Teflon™ cannula directed the gaseous stimulus from the olfactometer to the subject’s nose in the MRI-room. Table 2 lists the 5 sensory stimuli, including their origin and their concentrations.

**Table 2:** Sensory conditions, their origins and percentage (vol/vol) dilutions.

Conditions	Origin	Dilution
[CO_2_]	Praxair, Dresden, Germany	40% v/v
[Rose odor]	Phenyl ethyl alcohol Sigma. Aldrich Chemie GmbH, Riedstraße 2,Stauheim CAS # 60-12-8	20% v/v
[Orange odor]	Orange aroma oil; Frey and Lau, Henstedt-Ulzburg, Germany	20% v/v
[CO_2_+Rose odor]	As above	40% v/v +20%v/v
[CO_2_+Orange odor]	As above	40% v/v +20%v/v

### fMRI Experimental Paradigm

The study was performed on a 1.5 Tesla MR-scanner (Siemens Sonata, Erlangen, Germany). The experiment, which lasted approximately 60 min (from arrival to departure of the subject), comprised 5 functional sessions presented in a randomized order, one for each stimulus condition: [CO_2_ component], [Rose component], [Orange component], [pleasant mixture], [unpleasant mixture]. Each experimental session in turn comprised 6 on/off-block sub-sessions, with 30-sec blocks presented alternately in the On (stimulus-on) and Off (stimulus-off) conditions. The fMRI data were collected in 96 volumes/session with a 36 axial-slice matrix 2D SE/EP sequence (Matrix: 64×64; TR: 3 sec; TE: 35 ms; FA: 90°; voxel size: 3×3×3.75 mm). Session duration was 24 minutes. In the 6 minutes immediately following, a high-resolution T1-weighted image of the brain (3D IR/GR sequence: TR = 2180 ms/TE = 3.93 ms) was acquired.

During the scanning sessions, subjects were instructed to breathe through their mouth without concomitant nasal airflow (velopharyngeal closure, as described above), were not cued for any stimulus presentation and were not aware of the identity of stimuli during each experimental session. Moreover, they were not asked to perform any detection or cognitive task during stimulus presentation. After each session however, they were asked to evaluate the stimuli in terms of intensity (on a scale from “0”  =  “not perceived” to “10”  =  “extremely intense”) and of pleasantness (on a scale from “−5”  =  “extremely unpleasant” to “+5”  =  “extremely pleasant”), and they were also asked to identify the stimulus presented during each session.

### Data Analysis

fMRI data analysis used SPM8 software (Statistical Parametric Mapping; Wellcome Department of Cognitive Neurology, London, UK) implemented in Matlab 7.1 (MathWorks Inc., Natick, MA, USA). After spatial pre-processing (registering, realignment, co-registration between functional and structural images, normalization in a stereotaxic space, and smoothing by means of a 7*7*7 mm3 FWHM Gaussian kernel) [37], first-level statistical analysis was implemented with canonical hemodynamic response functions. Group analysis used a random-effects model [38]. Activation coordinates were presented in MNI space. A whole brain analysis was realized and loci of activations were identified using the Mai Atlas [39].

At the individual level, brain activation induced by the pleasant (or unpleasant) mixture was analyzed by comparing the activation pattern for each mixture condition and that obtained with their respective individual unimodal components (i.e., [CO_2_] and [Rose], or [CO_2_] and [Orange] depending on the subject). The resulting contrasts were then entered into a group analysis whereby they were compared to the no-stimulus baseline (averaged from all conditions). Areas of significant activation were identified at cluster level for values exceeding a p-value of 0.001 (3 voxels). Data were corrected for whole brain family-wise error. However, due to the conservative nature of the contrasts comparing the two mixtures, we established a level of significance of p<0.001 (uncorrected) with a cluster criterion of 3 voxels for the following contrasts: (a) ([pleasant mixture] vs. individual components) vs. ([unpleasant mixture] vs. individual components) and (b) ([unpleasant mixture] vs. individual components) vs. ([pleasant mixture] vs. individual components).

On a perceptual level, the number of correct stimulus identifications was counted for each condition and statistically compared using the Friedman Test. Practically, for conditions that included the odor stimuli or CO_2_ alone ([Rose], [Orange], [CO_2_+Rose], [CO_2_+Orange] and [CO_2_]), responses were counted as correct if the subject identified the source of the stimuli (i.e., rose, orange, carbon dioxide) or at least its semantic category (i.e., flower or citrus fruit for odors). For intensity and pleasantness ratings, a repeated ANOVA with compounds ([CO_2_], [Rose], [Orange], [pleasant mixture], [unpleasant mixture]) as a within-subjects factor was performed. If a significant main effect of compounds was observed, the analysis was followed by Tukey’s honest significance tests to control for multiple statistical comparisons. Moreover, difference in compound intensity between the pleasant mixture and the relatively unpleasant mixture was used as covariate in an ANCOVA with mixture pleasantness as the dependent variable in order to correct for the potential influence of stimulus intensity on pleasantness ratings between mixtures.
